# Fabrication of Lithium/Strontium‐Releasing Smart Bioactive Glasses with Anti‐Inflammatory and Osteogenic Effects Tailored to Pathological Stages

**DOI:** 10.1002/mabi.202500268

**Published:** 2025-06-29

**Authors:** Hirohiko Sakai, Jun‐Ichi Sasaki, Haruaki Kitagawa, Gabriela L. Abe, Tomoki Kohno, Naoya Funayama, Satoshi Imazato

**Affiliations:** ^1^ Department of Dental Biomaterials Graduate School of Dentistry The University of Osaka Suita Osaka Japan; ^2^ Joint Research Laboratory of Advanced Functional Materials Science Graduate School of Dentistry The University of Osaka Suita Osaka Japan

**Keywords:** anti‐inflammation, bone regeneration, pathological stage tracking, smart bioactive materials, stepwise ion release

## Abstract

Bioactive glasses (BGs) are highly biocompatible with affinity for hard tissues and exhibit high bioactivity through ion release. Smart BGs that allow controlled ion release are required because uncontrolled release can lead to unexpected adverse effects on tissue regeneration. Strontium promotes osteoblast differentiation of mesenchymal stem cells (MSCs) and inhibits osteoclast activity. In this study, the release profile of strontium is regulated by the incorporation of aluminum into a phosphate‐based BG. Furthermore, composites of strontium‐releasing BG and lithium‐releasing BG (Li/Sr‐BG) show stepwise ion release, with rapid lithium release followed by sustained strontium release. Li/Sr‐BG increases the expression of osteogenic markers and mineral deposition in MSCs, but suppresses osteoclast maturation, including multinucleation and osteoclast marker expression. Additionally, application of Li/Sr‐BG to inflammatory macrophages decreases phagocytic activity and inflammatory gene expression, while increasing the expression of anti‐inflammatory markers. Analysis of signaling proteins reveals that osteogenic and anti‐inflammatory effects of Li/Sr‐BG are attributed to the release of strontium and lithium, respectively. This study demonstrates that Li/Sr‐BGs can be used for the development of novel smart bioactive materials that effectively suppress inflammation and promote bone formation in a manner that follows the process of bone regeneration.

## Graphical abstract

Bioactive glasses with controllable dual ion release capability are developed to aid bone regeneration. The bioactive glasses rapidly release lithium ions that exert an anti‐inflammatory effect, suppressing inflammation after bone injury. Simultaneously, the bioactive glasses slowly release strontium ions, which support bone formation by suppressing osteoclast resorption and promoting osteoblast differentiation. Rapid lithium‐ion release is followed by sustained strontium‐ion release.

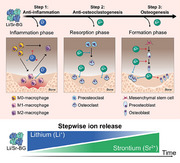

## 1 Introduction

Bioactive glasses (BGs) exhibit excellent biocompatibility and bioactivity and have recently attracted much attention in the orthopedic and dental fields [1]. BGs have a strong affinity with hard tissues and have been applied to hard tissue restoration in various craniofacial, maxillofacial, and periodontal repairs. BGs can act as carriers of ions, such as sodium (Na), phosphorus (P), and calcium (Ca), and can provide therapeutic functionality through the release of such ions [2]. In addition, advances in BG composition have led to the development of multifunctional BGs [2]. For example, silver‐releasing BGs show antibacterial properties [3], zinc‐loaded BGs promote angiogenesis [4], and a boron‐loaded BG displayed bone‐regenerating and angiogenic effects [5]. In addition, magnesium‐loaded BGs with antimicrobial and bone regenerative properties have been developed [6]. We have previously reported a lithium (Li)‐ releasable BG (Li‐BG) that has anti‐inflammatory effects [7]; Li‐BG regulates macrophage activity and suppresses local inflammation through the rapid release of Li, which has anti‐inflammatory properties.

Strontium (Sr), an alkaline earth metal with similar chemical properties to Ca, acts as an agonist for Ca‐sensing receptors, which promotes osteoblast differentiation of mesenchymal stem cells (MSCs) and inhibits osteoclast activity [8, 9]. In clinical applications, Sr administration can increase bone density and reduce the risk of bone fracture. This makes Sr formulations useful in the treatment of osteoporosis [10]. Additionally, Sr incorporation into an implant fixture optimized surface properties and facilitated osteogenic differentiation of MSCs [11]. Sasaki et al. reported that Sr‐releasable bone substitutes promoted the differentiation of osteoprogenitor cells and greatly enhanced bone formation [12]. In scaffold applications, Sr‐doped BGs have demonstrated bioactivity by stimulating mineralization, highlighting their potential for promoting bone repair and regeneration [13].

After injury, bone undergoes an inflammatory phase followed by a regenerative process [14]. During the inflammatory phase, M1 inflammatory macrophages are activated by cytokines, such as tumor necrosis factor alpha (TNF‐α), interferon gamma (IFN‐γ), and interleukin 6 (IL‐6) to remove pathogens and dead cells at the injury site [15]. Subsequently, M1 macrophages repolarize into M2 anti‐inflammatory macrophages [16]. Once the inflammation subsides, typically within a week, bone healing proceeds sequentially through bone resorption and bone formation phases [17]. During the resorption phase, monocytes and macrophages differentiate into osteoclasts, which secrete cathepsin K (CTSK) and tartrate‐resistant acid phosphatase (TRAP) [18]. This process facilitates the resorption of bone matrix over a period of 2–3 weeks [19]. In the bone formation phase, osteoblasts differentiated from MSCs adhere to freshly resorbed bone surfaces and initiate bone formation with the deposition of a non‐collagenous calcified matrix over a period of 4–5 weeks [20]. Bone healing is then achieved within 3–4 months through the secretion of a collagen‐containing bone matrix by osteoblasts [21].

**FIGURE 1:**
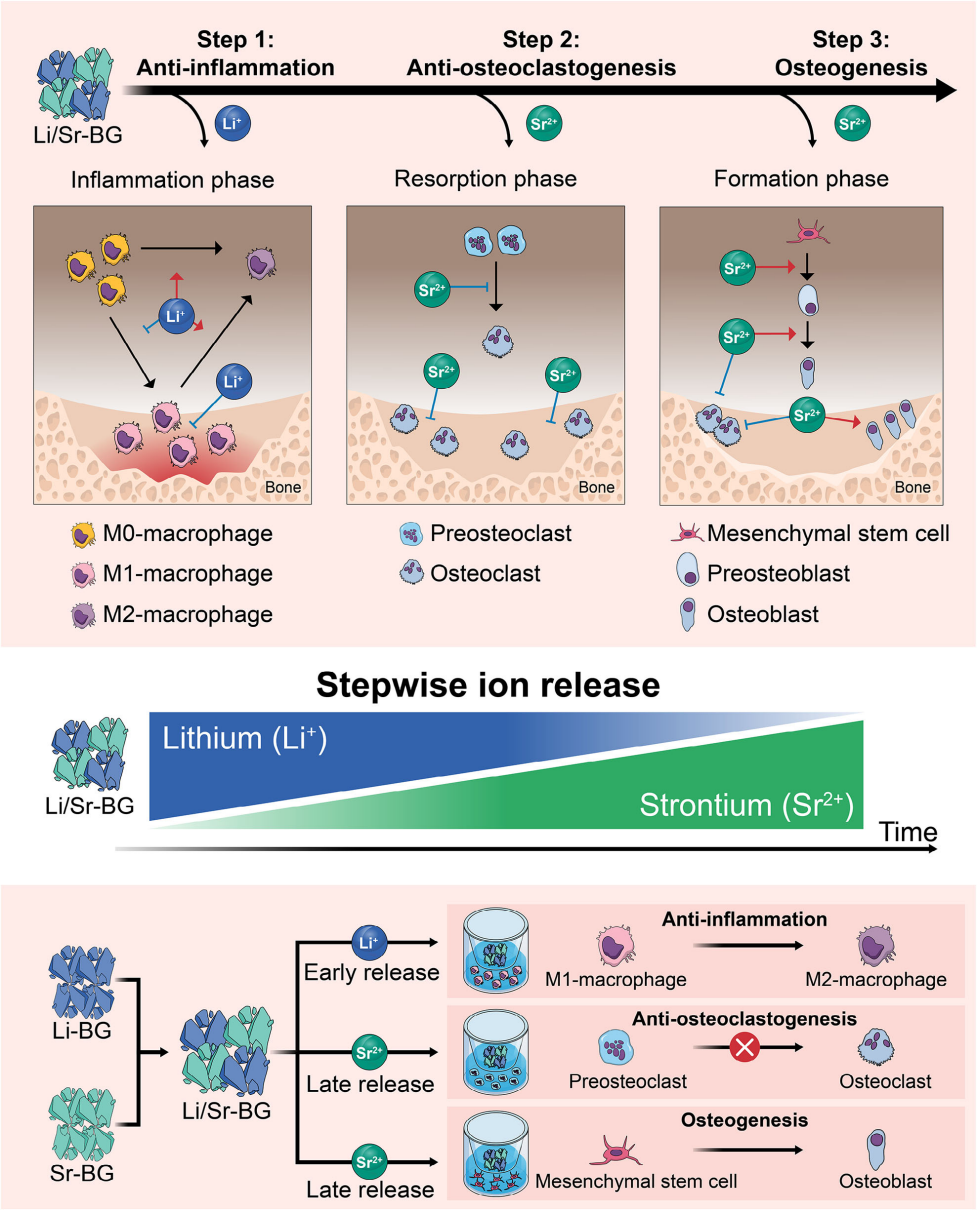
Schematic illustration of lithium/strontium releasing smart bioactive glass (Li/Sr‐BG). Li/Sr‐BG rapidly releases lithium ions that exert an anti‐inflammatory effect, suppressing inflammation after bone injury. Simultaneously, Li/Sr‐BG slowly releases strontium ions, which support bone formation by suppressing osteoclast resorption and promoting osteoblast differentiation. Cells were cultured with Li/Sr‐BGs using a Transwell insert, and the anti‐inflammatory and anti‐osteoclastogenic effects, and the osteogenic potential of Li/Sr‐BGs were evaluated in vitro.

Considering the above, we hypothesized that mixtures of Li‐BG and Sr‐releasable BG (Sr‐BG), which promote anti‐inflammation and bone formation, respectively, would lead to rapid and effective bone regeneration. Accordingly, BGs with a precisely controlled ion release profile are required to ensure the prompt supply of Li^+^ during the inflammatory phase and the long‐term release of Sr^2+^ during the formation phase. The aim of this study was to develop novel BGs that sequentially release Li^+^ and Sr^2+^ as next‐generation smart bioactive materials that follow pathological stages. We fabricated four Sr‐BGs with different solubilities by incorporating aluminum (Al), which acts as a glass network‐forming agent. Additionally, we evaluated the ion release profiles and biological effects of Sr‐BGs and composites of Sr‐BG and Li‐BG (Figure 1).

## 2 Experimental Section

### 2.1 Materials

Ammonium dihydrogen phosphate (NH_4_H_2_PO_4_), sodium carbonate (Na_2_CO_3_), calcium carbonate (CaCO_3_), strontium carbonate (SrCO_3_), lithium carbonate (Li_2_CO_3_), and aluminum oxide (Al_2_O_3_) were mixed according to the compositions in Table 1. These ingredients were melted at 1100 °C for 2 h and subsequently quenched on a steel plate to form Sr‐BGs (Sr‐Al0, Sr‐Al2, Sr‐Al4, Sr‐Al6) and Li‐BG. The experimental BGs were ground in an alumina ball mill at 100 rpm for 30 min. The Li‐BG and each Sr‐BG were mixed at a weight ratio of 1:1 and labeled as Li/Sr‐BGs (Li/Sr‐Al0, Li/Sr‐Al2, Li/Sr‐Al4, Li/Sr‐Al6).

**TABLE 1:** Raw materials and weight ratios (wt.%) used for fabricating bioactive glasses.

	NH_4_H_2_PO_4_	Na_2_CO_3_	CaCO_3_	SrCO_3_	Li_2_CO_3_	Al_2_O_3_
Sr‐Al0	58.7	12.0	/	29.3	/	/
Sr‐Al2	58.7	10.8	/	29.3	/	1.2
Sr‐Al4	58.7	9.6	/	29.3	/	2.4
Sr‐Al6	58.7	8.4	/	29.3	/	3.6
Li‐BG	67.3	/	11.1	/	21.6	/

### 2.2 Characterizations of Experimental BGs

To verify the morphology and size of the obtained BG particles, they were assessed by scanning electron microscopy (JSM‐6390; JEOL, Tokyo, Japan) and laser diffraction particle size analysis (Partica LA‐960V2; Horiba, Kyoto, Japan). The specific surface area of each Sr‐BG was determined by the Brunauer–Emmett–Teller (BET) method using a surface area measuring system (Belsorp II mini; MicrotracBEL, Osaka, Japan). Fourier‐transform infrared (FT‐IR) spectroscopy was carried out to investigate the chemical structures of BGs. The BG particles were mixed with potassium bromide and loaded on a sample cup as a flat loose powder. FT‐IR spectra were obtained by powder diffuse reflectance using an FT‐IR spectrophotometer (FTIR‐8300; Shimadzu, Kyoto, Japan). Fifty scans were collected with a range of 1400 to 600 cm^−1^ at a resolution of 1 cm^−1^. Crystallinity of BGs was evaluated by X‐ray diffraction (RINT‐2000; Rigaku, Tokyo, Japan). Diffraction patterns were recorded from 10° to 70° 2*θ* using Cu‐Kα radiation. The distribution and composition of BG elements were investigated by energy‐dispersive X‐ray spectroscopy (JSM‐F100; JEOL) and X‐ray fluorescence spectrometry (ZSX‐Primus IV; Rigaku), respectively.

All experimental BGs were sterilized with ethylene oxide gas and stored in dry cabinets at room temperature for further use.

### 2.3 Solubility and Ion Release Profiles of BGs

The solubility of experimental BGs was evaluated by gravimetry of residual BGs in water. Briefly, BG particles (50 mg) were immersed in distilled water (10 mL) at 37 °C for up to 100 days. The BG particles were filtered through a 0.22 µm syringe filter (Merck Millipore, Darmstadt, Germany) and then dried at 60 °C for 48 h. The ratio of residual BG was calculated according to the equation:

(1) $$\mathrm{Residual}\;\mathrm{BG}\left(\%\right)=\left(W_{2}-W_{1}\right)/50\times 100$$

where *W_1_* is the weight of syringe filter; *W_2_* is the total weight of the undissolved particles and the syringe filter, respectively (*n* = 4).

To determine the release profiles of Sr^2+^ and Li^+^ from experimental BGs, Sr‐BGs (1 mg), Li‐BG (1 mg), and Li/Sr‐BGs (2 mg) were placed in 12‐well Transwell inserts of 0.4 µm‐pore size (Corning, Corning, NY, USA). The inserts were then immersed in Alpha‐Minimum Essential Medium (αMEM) (2 mL; Wako, Osaka, Japan), and kept at 37 °C for up to 100 days. The concentrations of Sr^2+^ and Li^+^ were measured using an inductively coupled plasma‐optical emission spectrometer (iCAP7200; Thermo Fisher Scientific, Waltham, MA, USA) (*n* = 4). The medium was replaced with fresh αMEM on the day of measurement.

### 2.4 Cell Culture

Human bone marrow‐derived mesenchymal stem cells (BMSCs) (Lonza, Basel, Switzerland) were cultured in αMEM containing 20% fetal bovine serum (FBS) (Nichirei, Tokyo, Japan) and 1% penicillin/streptomycin (Sigma Aldrich, St. Louis, MO, USA). RAW 264.7 murine macrophages (Riken Cell Bank, Saitama, Japan) were cultured in αMEM supplemented with 10% FBS and 1% penicillin/streptomycin [22]. The cells were maintained at 37 °C in a humidified 5% CO_2_ incubator and passaged at 80% confluency. The cells from passages 3 to 8 were used for the experiments. Osteoclast differentiation was induced in RAW 264.7 cells by culture in growth medium containing recombinant murine receptor activator of nuclear factor kappa B (NF‐κB) ligand (RANKL) (50 ng mL^−1^; PeproTech, Rocky Hill, CT, USA) for 24 h. M1 polarization of RAW 264.7 cells was induced by incubation with growth medium supplemented with lipopolysaccharides (LPS) (100 ng mL^−1^; Sigma‐Aldrich) and recombinant mouse IFN‐γ (20 ng mL^−1^; BioLegend, San Diego, CA, USA) for 24 h.

### 2.5 Cytotoxicity

The cytotoxicity of experimental BGs on BMSCs and macrophages was evaluated in accordance with ISO 10993–5 [23]. Briefly, Sr‐BGs (10 mg) or Li/Sr‐BGs (20 mg) were immersed in culture medium (10 mL) at 37 °C and extracts were collected after 24 h. Next, BMSCs and RAW 264.7 cells (2.5 × 10^3^ cells cm^−2^) were cultured in 96‐well plates for 24 h. After washing with phosphate buffered saline (PBS), the cells were cultured for another 24 h with the extracts (100%) or with serial dilutions of the extracts in culture medium (50%, 25%, 12.5%, and 6.25%). Cell viability was measured using the WST‐8 assay (Dojindo, Kumamoto, Japan). The colorimetric absorbance of each well at 450 nm was measured using a microplate reader (iMark; Bio‐Rad, Hercules, CA, USA) (*n* = 6). The blank and medium controls were treated identically. The results were compared with those of cells cultured using fresh medium without extract (0%).

### 2.6 Cell Proliferation

BMSCs (2.5 × 10^3^ cells cm^−2^) and M1 macrophages (2.5 × 10^4^ cells cm^−2^) were seeded in 12‐well plates and cultured for 24 h. Sr‐BGs (1 mg) and Li/Sr‐BGs (2 mg) were then applied to the cells using a Transwell insert with culture medium (2 mL). BMSC and M1 macrophage numbers were assessed by WST‐8 assays for up to 7 days. The absorbance of each well at 450 nm was measured using an iMark microplate reader (Bio‐Rad) (*n* = 6). BMSCs cultured without BGs were used as controls. M1 macrophages without BG treatment and M0 macrophages without LPS induction served as M1 and M0 controls, respectively. The numbers of BMSCs and macrophages were normalized against the controls on day 1 and day 3, respectively.

### 2.7 Gene Expression

The impact of experimental BGs on gene expression in BMSCs and macrophages was investigated by real‐time reverse transcription‐polymerase chain reaction (RT‐PCR). BMSCs and M1 macrophages were cultured with Sr‐BGs (1 mg) and Li/Sr‐BGs (2 mg) for 7 and 14 days, and 1 and 3 days, respectively. Osteoclasts were cultured with Li/Sr‐BG (2 mg) for 1 and 3 days. Experimental BGs were applied to these cells using Transwell inserts. The mRNA levels of *Runt‐related transcription factor 2* (*RUNX2*), *Alkaline phosphatase* (*ALP*), *Osteoprotegerin* (*OPG*), and *Glyceraldehyde 3‐phosphate dehydrogenase* (*GAPDH*) in BMSCs; *Ctsk*, *Trap*, and *Gapdh* in osteoclasts derived from RAW 264.7 cells; and *Tnf*‐α, *Il‐6*, *Nitric oxide synthase 2* (*Nos2*), *Arginase 1* (*Arg1*), *Mannose receptor C‐type 1* (*Mrc1*), *Early growth response 2* (*Egr2*), and *Gapdh* in macrophages were evaluated using the TaqMan Gene Expression Cells‐to‐Ct kit (Thermo Fisher Scientific). mRNA levels relative to those of GAPDH were calculated using the 2^−ΔΔCt^ method (*n* = 6). BMSCs cultured without BGs were used as controls. M0 macrophages without RANKL stimulation and osteoclasts not treated with BGs were used as negative controls and positive controls, respectively. M1 macrophages cultured without BGs and M0 macrophages without LPS stimulation were designed as M1 and M0 controls, respectively.

### 2.8 ALP Activity

BMSCs were cultured with Sr‐BG (1 mg) or Li/Sr‐BG (2 mg) using a Transwell insert and ALP activity was measured after 7 and 14 days using a TRACP & ALP assay kit (Takara Bio, Kusatsu, Japan). Briefly, BMSCs were washed three times with PBS, then 0.1% NP‐40 (Takara Bio) was added to each well, followed by a 5‐min incubation. Subsequently, p‐nitrophenyl phosphate was applied to each well and incubated at 37 °C for 60 min. The absorbance of each specimen at 410 nm was measured using an iMark microplate reader (Bio‐Rad) (*n* = 6). ALP activity was corrected against total protein amount, which was measured using a dye reagent (Bio‐Rad). BMSCs cultured without experimental BGs were used as the control.

### 2.9 Mineral Deposition

BMSCs were cultured with Sr‐BG (1 mg) or Li/Sr‐BG (2 mg) using a Transwell insert and the deposition of mineralized matrices was visualized by von Kossa staining. The cells were washed with PBS and fixed with 4% paraformaldehyde after 21 and 28 days of culture. The fixed cells were then washed three times with distilled water, followed by the addition of 5% silver nitrate aqueous solution (Sigma‐Aldrich) and ultraviolet light exposure for 30 min (*n* = 6). The stained samples were imaged using a digital camera (D5500; Nikon, Tokyo, Japan). Mineralized nodule formation was semi‐quantified using ImageJ software (NIH, Bethesda, MD, USA). BMSCs cultured without experimental BGs were used as the control.

### 2.10 TRAP Activity

The effect of experimental BGs on osteoclast activity was evaluated by staining and quantification. Osteoclasts derived from RAW 264.7 cells were cultured with Li/Sr‐BGs (2 mg) using a Transwell insert for 3 days. For TRAP staining, cells were fixed with 4% paraformaldehyde, washed three times with PBS, and then analyzed with a TRAP staining kit (Wako). Briefly, a solution of sodium tartrate mixed with acid phosphate was applied to the cells for 30 min at 37 °C. The stained cells were observed using an optical microscope (CK40; Olympus, Tokyo, Japan) equipped with a digital camera (DS‐Fi2; Nikon).

Next, TRAP activity in the osteoclasts described above was quantified using a TRACP & ALP assay kit (Takara Bio). The cells were lysed with NP‐40 (Takara Bio) and then incubated with sodium acetate solution containing p‐nitrophenyl phosphate and sodium tartrate at 37 °C for 60 min. The mixture was transferred to a 96‐well plate and the absorbance was measured at a wavelength of 410 nm using an iMark microplate reader (Bio‐Rad) (*n* = 6). TRAP activity was corrected against total protein amount, which was measured using a dye reagent (Bio‐Rad). M0 macrophages without RANKL stimulation and osteoclasts not treated with BGs were used as negative controls and positive controls, respectively.

### 2.11 Fluorescence Staining

Osteoclasts were cultured with Li/Sr‐BGs using Transwell inserts for 3 days, then fixed with 4% paraformaldehyde, and treated with 0.2% Tween‐20 (Nacalai Tesque, Kyoto, Japan). Cells were sequentially incubated in 2% bovine serum albumin for 10 min and washed three times with PBS. Actin fibers and cell nuclei were stained with rhodamine phalloidin (Thermo Fisher Scientific) and Hoechst 33342 (Thermo Fisher Scientific), respectively. The stained specimens were observed with a confocal laser microscope (TSC SP8; Leica, Wetzlar, Germany). M0 macrophages and untreated osteoclasts represent negative controls and positive controls.

### 2.12 Phagocytosis Assay

The activity of M1 macrophages was evaluated using a previously described phagocytosis assay [24]. Briefly, 3.0‐µm diameter polystyrene beads (Sigma‐Aldrich) were opsonized with human IgG (0.1 mg mL^−1^; Wako) at room temperature for 1 h. M1 macrophages (2.5 × 10^4^ cells cm^−2^) were cultured with Sr‐BGs (1 mg) or Li/Sr‐BGs (2 mg) for 24 h using a Transwell insert, and then opsonized beads (3.0 × 10⁵) were added to each well. After 1 h of culture, the cells were washed three times with PBS and fixed with 4% paraformaldehyde. The cells were stained with rhodamine phalloidin (Thermo Fisher Scientific) and Hoechst 33342 (Thermo Fisher Scientific) and then observed using a fluorescence microscope (TE2000‐U; Nikon). The phagocytic index and phagocytic efficiency of macrophages were calculated from the captured images using the following formulas:

(2) Phagocyticindex=Npb/Nc

(3) $$\mathrm{Phagocytic}\;\mathrm{efficiency}=\left(Npc/Nc\right)\times 100$$

where *Npb* is the number of phagocytosed beads; *Nc* is the number of cells in images; *Npc* is the number of phagocytosing cells, respectively (*n* = 6).

### 2.13 Western Blotting

BMSCs were cultured with Li/Sr‐BG (2 mg) using Transwell inserts for 14 days, while osteoclasts and M1 macrophages were treated with Li/Sr‐BG (2 mg) for 3 days. After washing with PBS, proteins were extracted from cells using RIPA lysis buffer (Cell Signaling Technology, Danvers, MA, USA). Then, protein (10 µg) was loaded and separated by 9% sodium dodecyl sulfate‐polyacrylamide gel electrophoresis and transferred to a nitrocellulose membrane (Bio‐Rad). The membranes were blocked with 5% skim milk solution for 30 min and incubated at 4 °C for 12 h with the following antibodies. For proteins extracted from BMSCs, anti‐calcineurin (Abcam, Cambridge, UK), anti‐nuclear factor of activated T cell 1 (NFATc1) (Santa Cruz, Dallas, TX, USA), and horseradish peroxidase (HRP)‐conjugated anti‐β‐actin (Proteintech, Rosemont, IL, USA); for proteins extracted from osteoclasts, anti‐NF‐κB (Cell Signaling Technology), anti‐cellular oncogene fos (c‐Fos) (Santa Cruz), anti‐NFATc1 (Santa Cruz), and anti‐β‐actin (Proteintech); for proteins extracted from M1 macrophages, anti‐glycogen synthase kinase‐3β (GSK‐3β) (Cell Signaling Technology), anti‐phospho‐GSK3β (p‐GSK‐3β) (Cell Signaling Technology), anti‐signal transducer and activator of transcription 3 (STAT3) (Santa Cruz), anti‐phospho‐STAT3 (p‐STAT3) (Santa Cruz), and anti‐β‐actin (Proteintech). Subsequently, HRP‐conjugated IgG secondary antibodies (Cell Signaling Technology) were applied to all primary antibodies except anti‐β‐actin at room temperature for 3 h. Immunoreactive bands were detected using a chemiluminescent reagent (Chemi‐Lumi One L; Nacalai Tesque) and an imaging device (iBright CL750; Thermo Fisher Scientific). Band images were saved in TIFF format and semi‐quantified using ImageJ (*n* = 6). BMSCs cultured without BGs were used as controls. M0 macrophages without RANKL stimulation and osteoclasts not treated with BGs were used as negative controls and positive controls, respectively. M1 macrophages cultured without BGs and M0 macrophages without LPS stimulation were designed as M1 and M0 controls, respectively.

### 2.14 Statistical Analysis

One‐way analysis of variance (ANOVA) with a Tukey or Dunnett post‐hoc test was used for comparisons among groups. A significant difference was reported for p‐values < 0.05. All statistical analyses were performed using SPSS software (IBM, Armonk, NY, USA). The normality of the data distribution within each experimental group was assessed by the Shapiro–Wilk test.

## 3 Results

### 3.1 Characteristics of Sr‐BGs and Li‐BG

**FIGURE 2:**
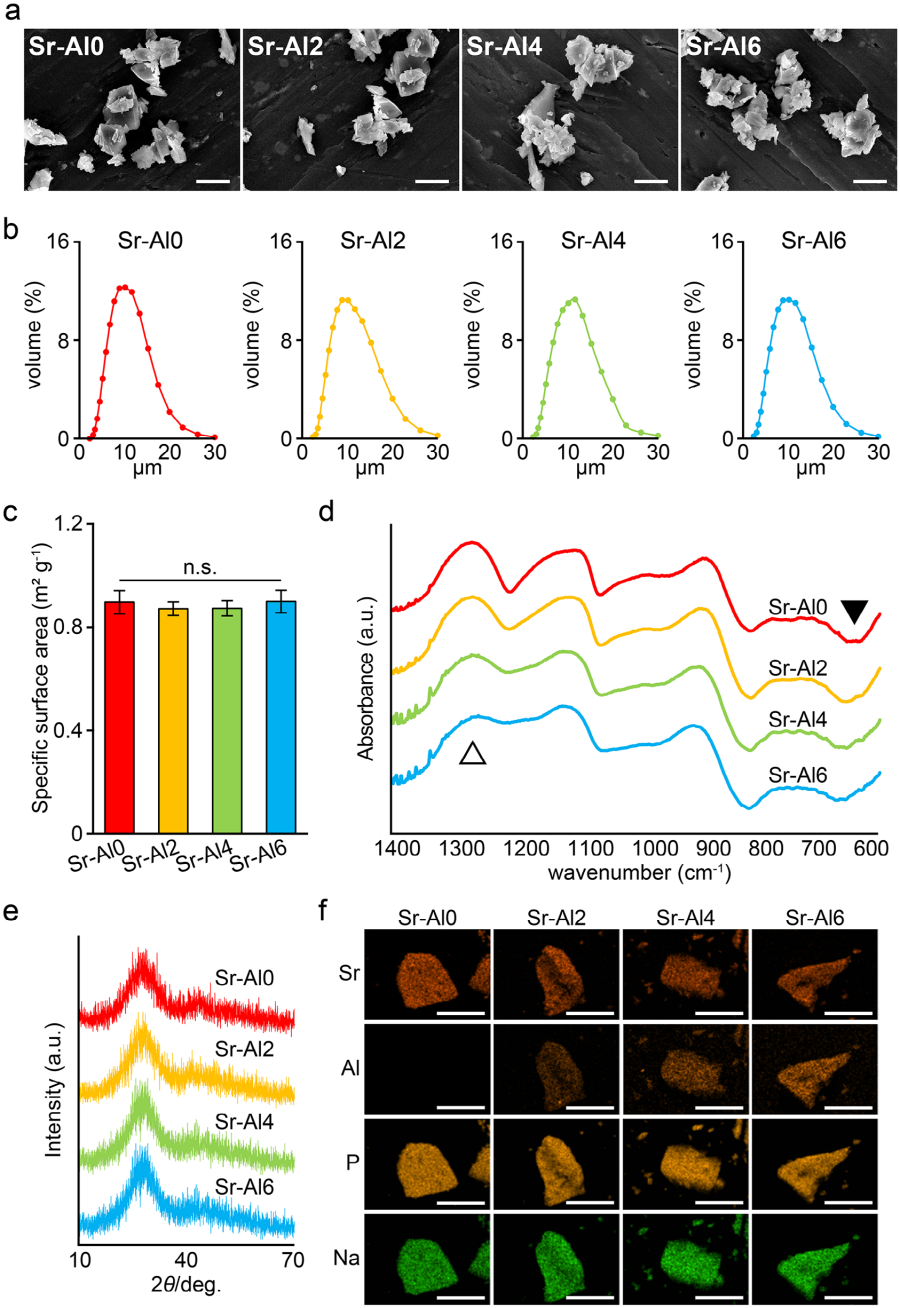
Characterization of Sr‐BGs. (a) Scanning electron microscopy images, (b) size distribution, (c) specific surface area, (d) FT‐IR spectra, and (e) X‐ray diffraction patterns of Sr‐BGs. FT‐IR spectra show bands for O‐P‐O bonds (1283 cm^−1^, open arrowhead) and P‐O‐Al bonds (600 to 650 cm^−1^, filled arrowhead). (f) Elemental mapping of Sr‐BGs particles showing the distribution of strontium (Sr), aluminum (Al), phosphorus (P), and sodium (Na). Scale bars = 10 µm.

Sr‐BG and Li‐BG particles were ≈10 µm along the major axis, exhibited an irregular polygonal shape, and showed no remarkable differences among the samples (Figure 2a,b, Figure S1a,b). The specific surface area of each Sr‐BG was ≈0.9 m^2^ g^−1^, and no significant differences were observed among the samples (Figure 2c). The FT‐IR spectra of Sr‐BGs are shown in Figure 2d. The band centered at 1283 cm^−1^, attributed to the asymmetric stretching vibration mode of the O‐P‐O bond [25], became narrower and decreased as the Al₂O₃ content increased. In addition, the low‐frequency band between 600 and 650 cm^−1^ shifted toward a higher wavenumber with increasing Al_2_O_3_ content, indicating the formation of P‐O‐Al bonds [26]. The X‐ray diffraction profiles of Sr‐BGs and Li‐BG showed a broad band, indicating a halo pattern with an amorphous phase (Figure 2e, Figure S1c). Energy‐dispersive X‐ray spectroscopy demonstrated that all constituent elements of Sr‐BGs were uniformly distributed within the particles (Figure 2f). X‐ray fluorescence spectrometry revealed that the ratio of Al_2_O_3_ increased in Sr‐BGs according to the raw material formulation, and that there were no differences in particle composition for SrO and P_2_O_5_ (Table 2).

**TABLE 2:** Composition of experimental bioactive glasses (mean ± SD mol%).

	P_2_O_5_	Na_2_O	CaO	SrO	Li_2_O	Al_2_O_3_
Sr‐Al0	43.8 ± 0.1	15.8 ± 0.3	/	40.4 ± 0.2	/	/
Sr‐Al2	43.5 ± 0.1	14.1 ± 0.1	/	39.9 ± 0.1	/	2.5 ± 0.1
Sr‐Al4	43.7 ± 0.1	12.3 ± 0.2	/	39.8 ± 0.1	/	4.1 ± 0.1
Sr‐Al6	42.9 ± 0.1	11.1 ± 0.2	/	40.1 ± 0.2	/	5.9 ± 0.1
Li‐BG	40.8 ± 0.4	/	17.0 ± 0.1	/	42.2 ± 0.4	/

**FIGURE 3:**
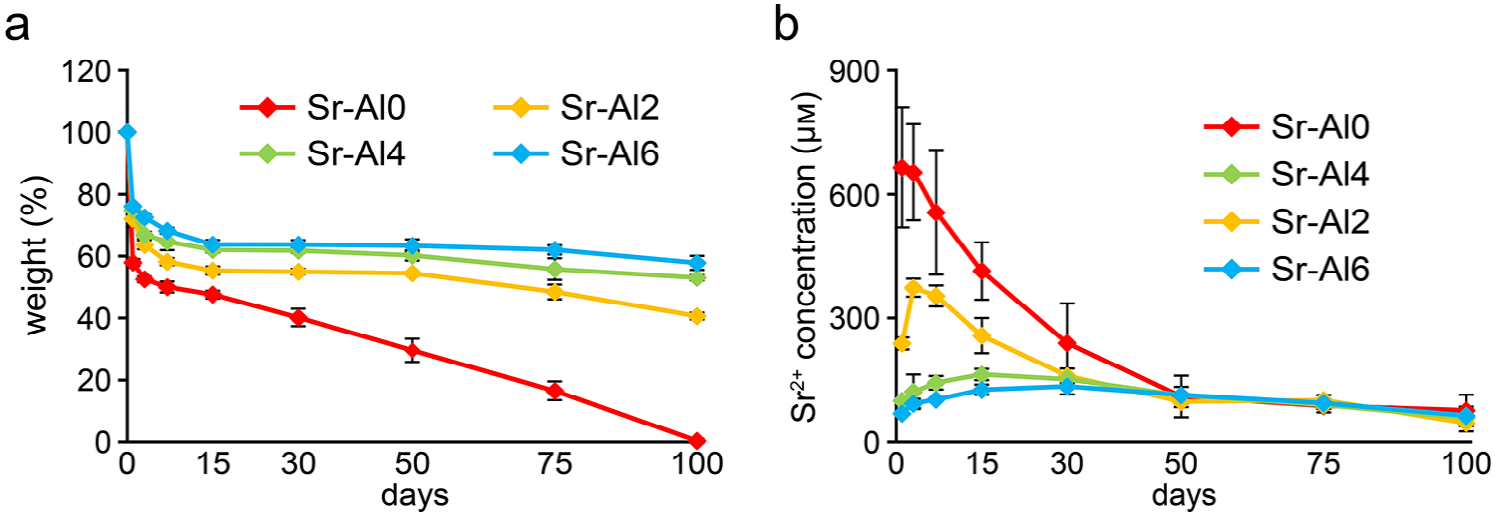
Solubility and ion release profiles of Sr‐BGs. (a) Residual rates and (b) Sr^2+^ release profiles of Sr‐BGs for 100 days (mean ± SD, *n* = 4).

The solubility of Sr‐BGs was investigated by determining their residual rate after immersion in water (Figure 3a). The residual rate of Sr‐BGs increased with an increasing Al ratio, indicating that the solubility decreased with increasing Al content. Sr‐Al0 exhibited the highest solubility among the Sr‐BGs and was completely dissolved by day 100. In contrast, Sr‐Al2, Sr‐Al4, and Sr‐Al6 remained 40.6 ± 1.1%, 53.0 ± 0.9%, and 57.8 ± 2.7% insoluble at day 100, respectively. regarding the ion release profile, all Sr‐BGs sustained Sr^2+^ release over 100 days (Figure 3b). The peak release of Sr^2+^ from Sr‐Al0 was observed on day 1; however, the time to maximum concentration was prolonged with increasing Al content. The maximum concentration of Sr^2+^ was released by Sr‐Al2 on day 3, by Sr‐Al4 on day 15, and by Sr‐Al6 on day 30. After reaching the maximum Sr^2+^ concentration, the release of Sr^2+^ gradually decreased during the experimental period for all Sr‐BGs. Li‐BG exhibited an initial burst of Li^+^ release, reaching ≈3.0 mm on day 1, followed by a gradual decline over a period of up to 100 days (Figure S1d).

### 3.2 Solubility and Ion Release Profiles of Li/Sr‐BGs

**FIGURE 4:**
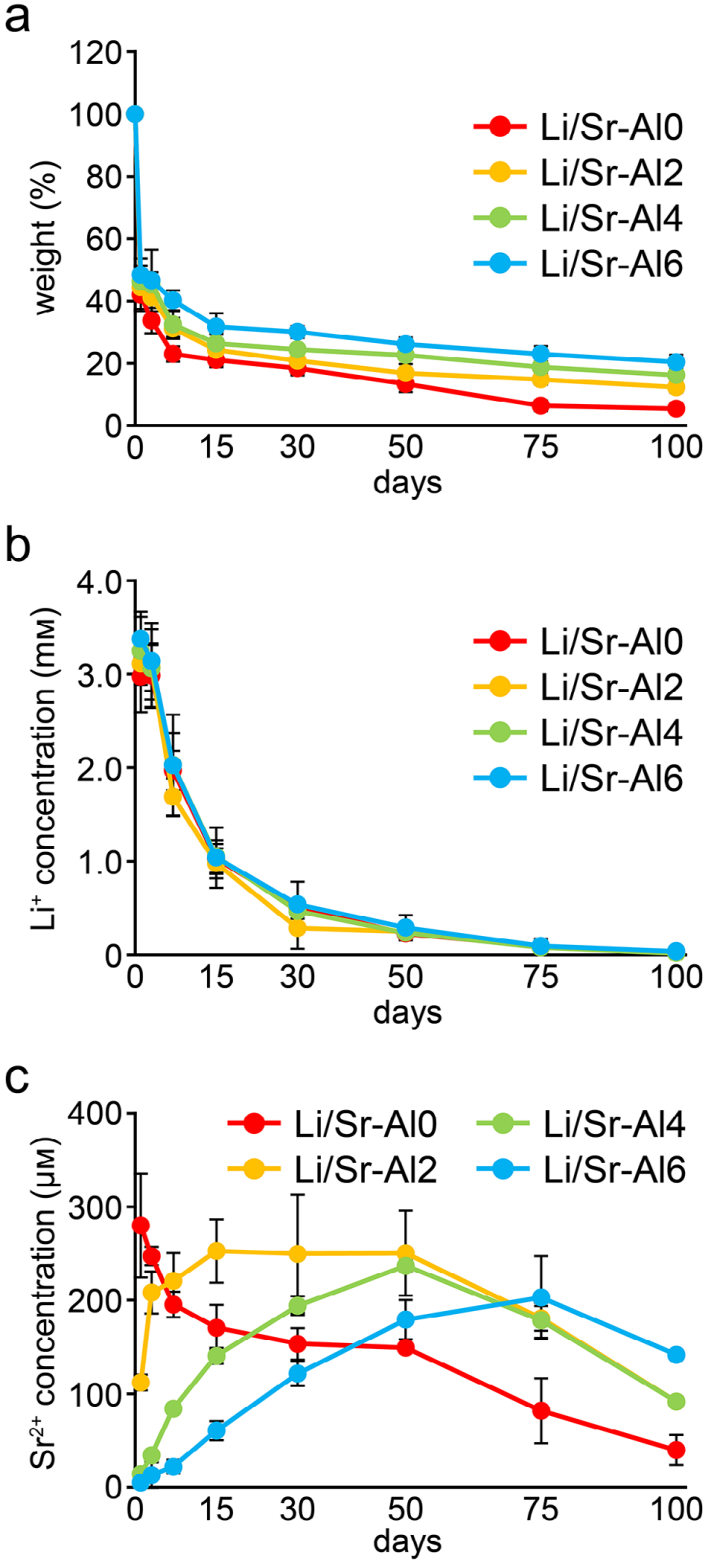
Solubility and ion release profiles of Li/Sr‐BGs. (a) Residual rates and release profiles of (b) Li^+^ and (c) Sr^2+^ from Li/Sr‐BGs for 100 days (mean ± SD, *n* = 4).

The residual rate of insoluble Li/Sr‐BGs increased with increasing Al content (Figure 4a). After immersion in water for 100 days, 5.4 ± 0.9% of Li/Sr‐Al0 remained while 20.4 ± 0.2% of Li/Sr‐Al6 remained. The Li/Sr‐BGs showed similar release profiles with an initial burst of Li^+^ release (Figure 4b). The concentration of Li^+^ was ≈3.0 mm on day 1, then gradually decreased for up to 100 days. Meanwhile, Li/Sr‐BGs exhibited different Sr^2+^ release profiles depending on the amount of Al incorporated (Figure 4c). Li/Sr‐Al0 released 279.9 ± 55.4 µm of Sr^2+^ on day 1, followed by a sustained reduction for up to 100 days. As with the Sr‐BGs, the period to maximum Sr^2+^ concentration was gradually prolonged in accordance with the Al ratio, with Li/Sr‐Al6 showing a peak concentration of Sr^2+^ release at day 75.

### 3.3 Effects of Sr‐BGs on BMSCs

**FIGURE 5:**
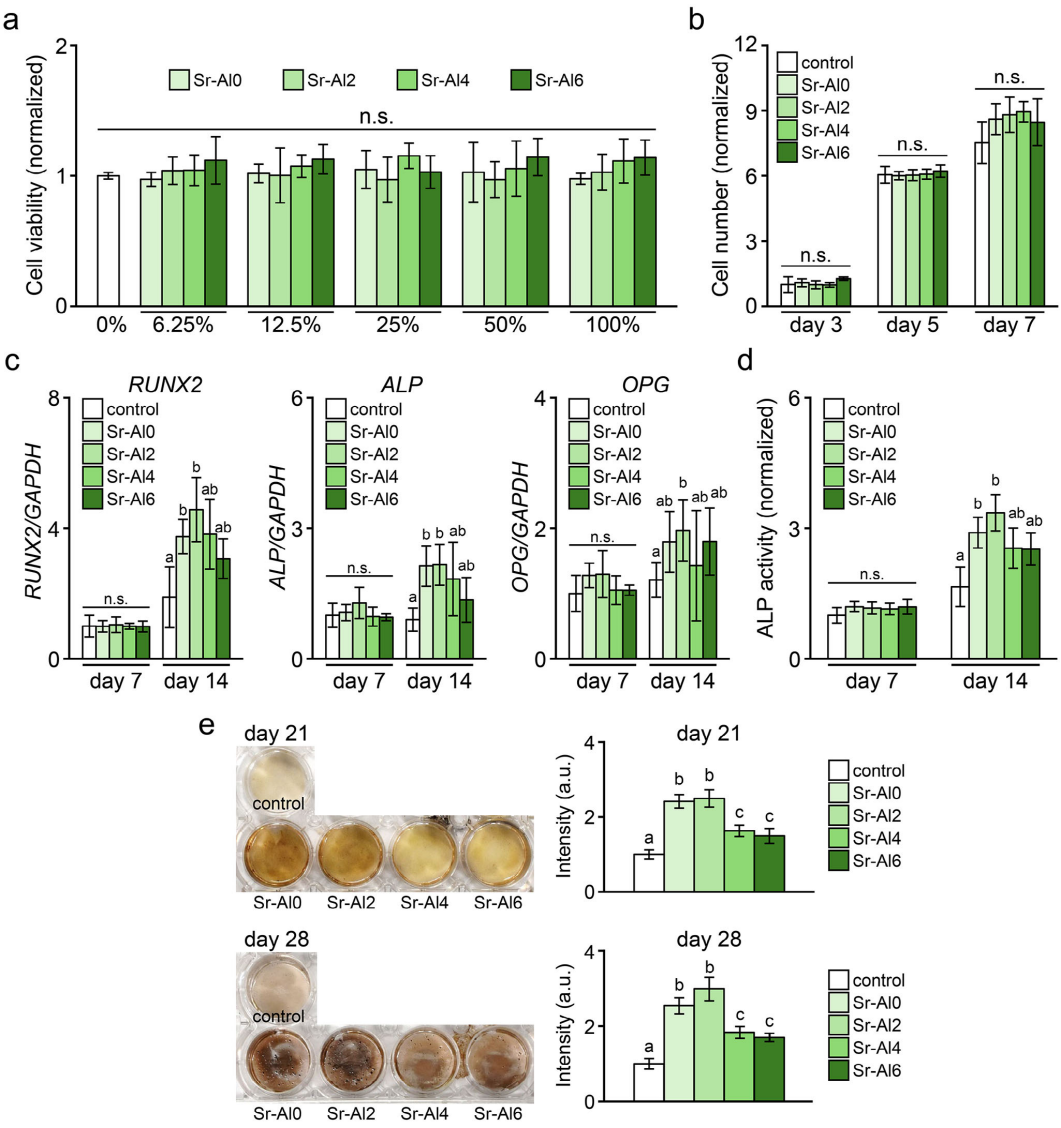
Effects of Sr‐BGs on BMSCs. (a) Cell viability and (b) proliferation of BMSCs treated with Sr‐BGs. (c) mRNA levels of osteogenic markers (*RUNX2*, *ALP*, *OPG*) and (d) ALP activity in BMSCs cultured with Sr‐BGs for 7 and 14 days. (e) Mineral deposition in BMSCs cultured with Sr‐BGs. Semi‐quantitative evaluation graphs of mineral matrix are shown on the right. BMSCs cultured without Sr‐BGs are shown as the control. Letters (a, b, c) indicate significant differences between groups (*p* < 0.05). Mean ± SD, *n* = 6, n.s.: not significant.

The cytotoxicity of Sr‐BGs on BMSCs was assessed using extracts (Figure 5a). Cells cultured with pure extracts (100%) and serially diluted extracts (50%–6.25%) of all Sr‐BGs demonstrated no significant differences in viability compared with cells in fresh medium (0%). Regardless of Al content, Sr‐BGs exerted no significant influence on BMSC proliferation for 7 days (Figure 5b). Expression of genes specifically related to osteogenic differentiation is presented in Figure 5c. On day 7, the expression of osteogenic markers showed no significant difference among the specimens; however, the expression levels of *RUNX2* and *ALP* were higher in the BMSCs treated with Sr‐Al0 and Sr‐Al2 than in controls on day 14. The expression of *OPG* on day 14 was significantly higher only in the cells cultured with Sr‐Al2 compared with the control.

The ALP activity of BMSCs was not significantly different with Sr‐BG treatment on day 7, but was upregulated in cells cultured with Sr‐Al0 and Sr‐Al2 compared with the control on day 14 (Figure 5d). Mineralized matrices formed by BMSCs were visualized by von Kossa staining (Figure 5e). On day 21 and 28, the cells treated with Sr‐BGs exhibited significantly more mineral deposition compared with the controls. In addition, the amount of mineralized matrix in BMSCs treated with Sr‐Al0 and Sr‐Al2 was significantly more than that in control cells and in cells treated with other Sr‐BGs.

### 3.4 Effects of Li/Sr‐BGs on BMSCs

**FIGURE 6:**
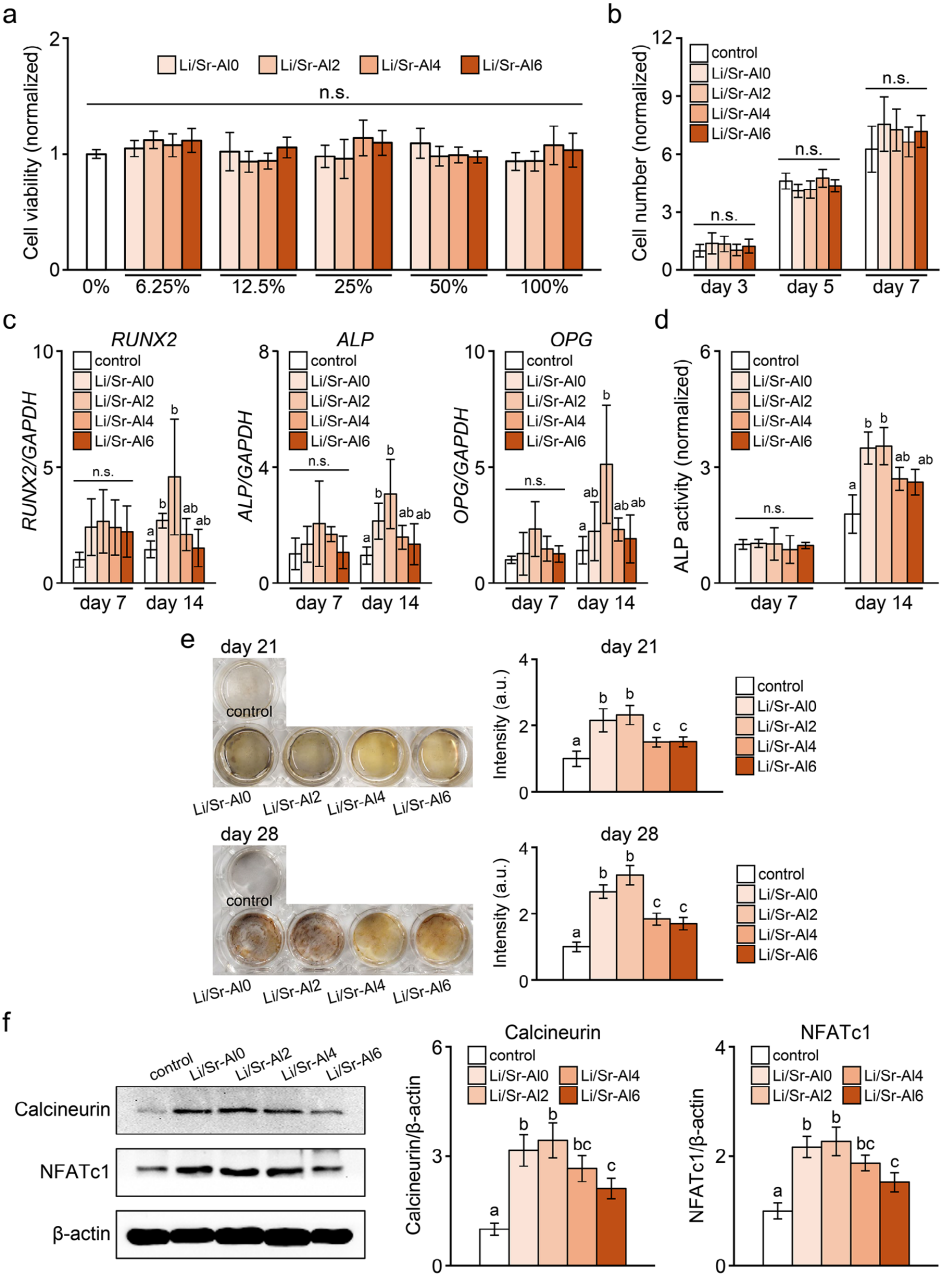
Effects of Li/Sr‐BGs on BMSCs. (a) Cell viability and (b) proliferation of BMSCs treated with Li/Sr‐BGs. (c) mRNA levels of osteogenic markers (*RUNX2*, *ALP*, *OPG*) and (d) ALP activity of BMSCs cultured with Li/Sr‐BGs for 7 and 14 days. (e) Mineral deposition in BMSCs cultured with Li/Sr‐BGs. (f) The protein levels of osteogenic signaling molecules in BMSCs cultured with Li/Sr‐BGs at day 14. Semi‐quantitative evaluation graphs of mineral matrix and protein levels are shown on the right. BMSCs cultured without Li/Sr‐BGs are shown as the control. Letters (a, b, c) indicate significant differences between groups (*p* < 0.05). Mean ± SD, *n* = 6, n.s.: not significant.

Cytotoxicity test results for Li/Sr‐BGs are presented in Figure 6a. The viabilities of cells cultured with pure (100%) and serially diluted extracts (50%–6.25%) were comparable with those of cells cultured with fresh medium (0%). As with the Sr‐BGs, none of the Li/Sr‐BGs had an impact on the proliferation of BMSCs (Figure 6b).

The expression levels of osteogenic differentiation markers showed no significant differences among BMSCs cultured with any Li/Sr‐BG on day 7 (Figure 6c). However, on day 14, *RUNX2* and *ALP* expression levels were notably elevated with Li/Sr‐Al0 and Li/Sr‐Al2 compared with the control. Furthermore, BMSCs treated with Li/Sr‐Al2 demonstrated a significant increase in *OPG* expression compared with the control on day 14. ALP activity assays demonstrated no significant differences between BMSCs cultured with Li/Sr‐BGs and control cells on day 7, while cells cultured with Li/Sr‐Al0 and Li/Sr‐Al2 showed significantly higher ALP activities on day 14 (Figure 6d). Cells treated with Li/Sr‐BGs exhibited a significant increase in the deposition of mineralized matrix compared with the control cells after 21 and 28 days of culture (Figure 6e). Meanwhile, cells cultured with Li/Sr‐Al0 and Li/Sr‐Al2 showed significantly greater amounts of mineralized matrix compared with cells cultured with other Li/Sr‐BGs.

The expression of osteogenic signaling molecules was evaluated by western blotting after 14 days of culture (Figure 6f). The levels of calcineurin and NFATc1 were significantly increased in BMSCs cultured with Li/Sr‐BGs compared with those in control cells. Notably, cells treated with Li/Sr‐Al0 and Li/Sr‐Al2 exhibited significantly higher levels of calcineurin and NFATc1 compared with cells treated with Li/Sr‐Al6.

### 3.5 Effects of Li/Sr‐BGs on Osteoclasts

**FIGURE 7:**
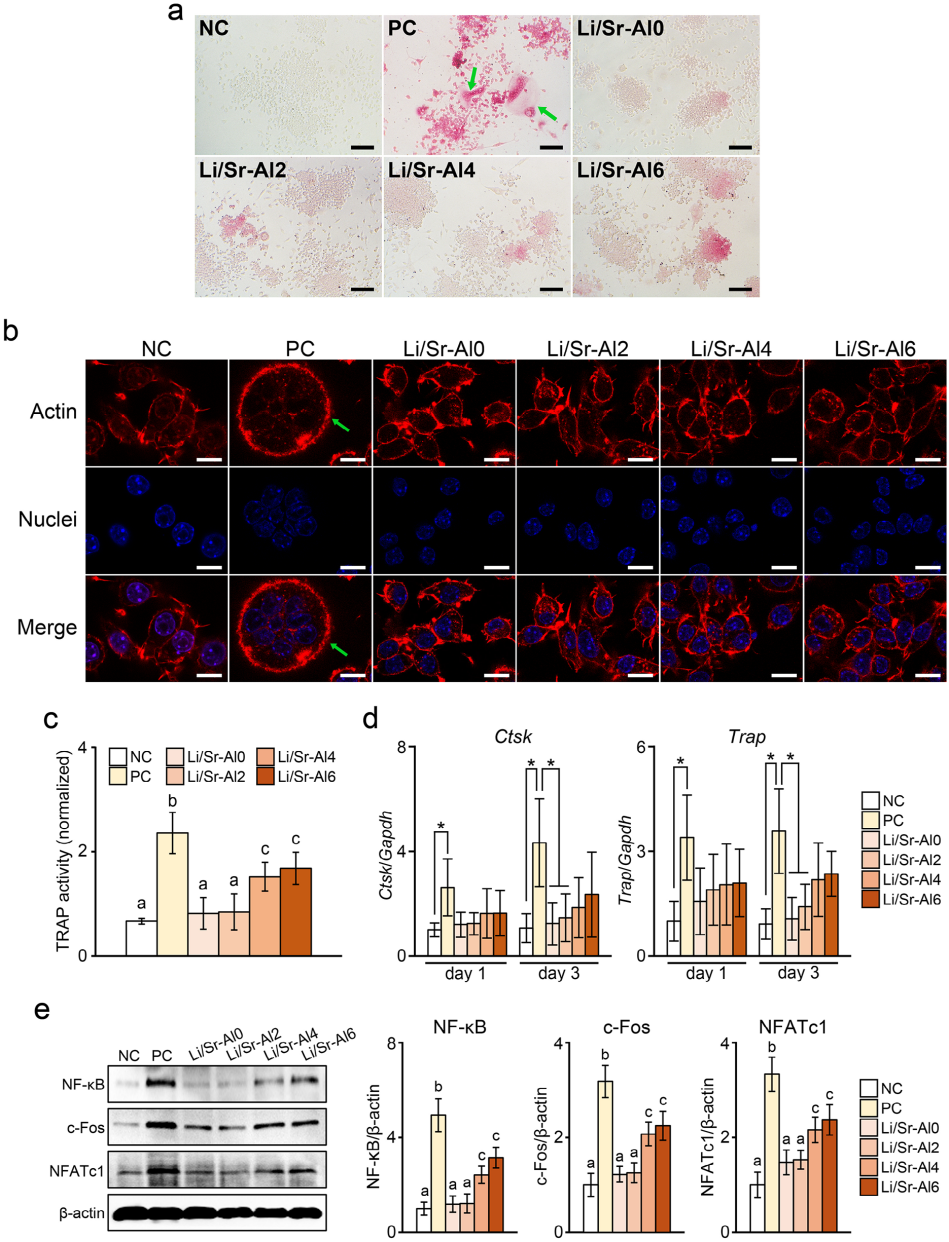
The effects of Li/Sr‐BGs on osteoclast maturation. (a) TRAP staining and (b) fluorescence staining of osteoclasts cultured with Li/Sr‐BGs for 3 days. Green arrows indicate multinucleated giant cells. (c) Quantitative assessment of TRAP activity in osteoclasts after 3 days of culture with Li/Sr‐BGs. (d) mRNA levels of osteoclastogenic markers (*Ctsk*, *Trap*) in osteoclasts treated with Li/Sr‐BGs on day 1 and day 3. (e) The protein levels of osteoclastic signaling molecules in osteoclasts cultured with Li/Sr‐BGs on day 3. Semi‐quantitative evaluation graphs of protein levels are shown on the right. M0 macrophages without RANKL stimulation and osteoclasts not treated with BGs are shown as negative (NC) and positive controls (PC), respectively. Letters (a, b, c) and asterisks indicate significant differences between groups (*p* < 0.05). Mean ± SD, *n* = 6, n.s.: not significant. Scale bars: (a) 10 µm; (b) 50 µm.

The effect of Li/Sr‐BGs on osteoclast maturation was evaluated in RANKL‐stimulated macrophages. On day 3, TRAP staining of positive control cells showed the presence of numerous TRAP‐positive multinucleated giant cells, whereas no multinucleated cells were detected in cells cultured with Li/Sr‐BGs or in negative control cells (Figure 7a). Fluorescence imaging revealed multinucleated positive control cells with actin‐rich structures; however, the formation of osteoclast‐like morphology was inhibited by Li/Sr‐BG treatment (Figure 7b).

TRAP quantification on day 3 further demonstrated that positive control cells exhibited significantly higher TRAP activity compared with the other groups (Figure 7c). Additionally, cells cultured with Li/Sr‐Al0 or Li/Sr‐Al2 showed TRAP activity levels that were significantly lower than those of cells cultured with the other Li/Sr‐BGs, and that were not significantly different compared with negative control cells. On day 1, the expression levels of osteoclast‐related markers, *Ctsk* and *Trap*, were not significantly different between cells treated with Li/Sr‐BGs and positive control cells (Figure 7d). However, on day 3, these levels were significantly reduced in cells cultured with Li/Sr‐Al0 or Li/Sr‐Al2 compared with positive control cells and were comparable to those of negative controls.

The effects of Li/Sr‐BGs on the levels of osteoclastic signaling molecules were evaluated after 3 days of culture (Figure 7e). Cells cultured with Li/Sr‐BGs exhibited significantly decreased levels of NF‐κB, c‐Fos, and NFATc1 compared with the positive control cells. Notably, no significant differences in protein levels were observed between cells treated with Li/Sr‐Al0 or Li/Sr‐Al2 and negative control cells.

### 3.6 Effects of Experimental BGs on Macrophages

**FIGURE 8:**
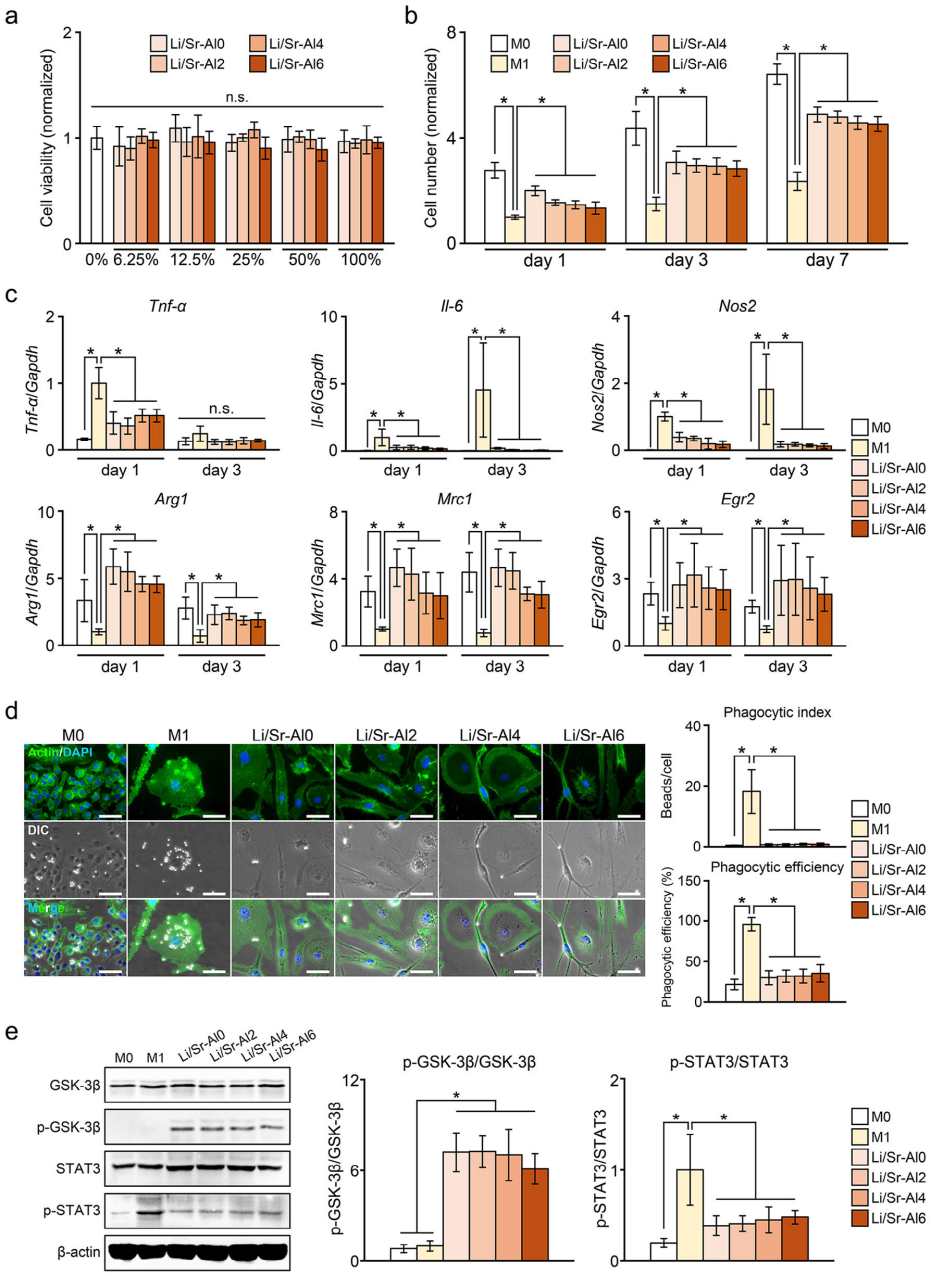
The effects of Li/Sr‐BGs on macrophages. (a) Cell viability and (b) proliferation of macrophages treated with Li/Sr‐BGs. (c) mRNA levels of inflammatory markers (*Tnf‐α*, *Il‐6*, *Nos2*) and anti‐inflammatory markers (*Arg1*, *Mrc1*, *Egr2*) in macrophages cultured with Li/Sr‐BGs on day 1 and day 3. (d) Photomicrographs of macrophages engaged in phagocytosis and corresponding semi‐quantitative analyses of phagocytic activity. (e) The levels of inflammation‐related proteins in macrophages cultured with Li/Sr‐BGs for 3 days. M0 macrophages and M1 macrophages cultured without BG are presented as M0 and M1, respectively. Asterisks indicate significant differences between groups (*p* < 0.05). Mean ± SD, *n* = 6, n.s.: not significant. Scale bars: 30 µm.

M0 macrophages were cultured for 24 h with extracts derived from Li/Sr‐BGs. Across all tested samples and dilutions, no significant difference in cell number was observed compared with cells cultured in fresh medium (0%) (Figure 8a). The number of proliferative M1 macrophages cultured without BG was significantly lower than the number of M0 macrophages on days 1, 3, and 7 (Figure 8b, Figure S2). There was no difference in cell numbers between Sr‐BG‐treated cells and M1 macrophages during the experimental period. In contrast, cells cultured with Li/Sr‐BGs had significantly restored proliferative capacity compared with M1 macrophages.

Cells cultured with Li/Sr‐BGs exhibited significantly reduced gene expression levels of *Tnf‐α*, *Il‐6*, and *Nos2* on day 1 compared with M1 (Figure 8c). Alternatively, the expression levels of anti‐inflammatory markers (*Arg1*, *Mrc1*, *Egr2*) in M1 macrophages were significantly increased by treatment with Li/Sr‐BGs. However, the expression levels of both inflammatory and anti‐inflammatory genes in Sr‐BG‐treated cells were not different compared with those in M1 cells (Figure S3).

The phagocytic activity of macrophages was evaluated using opsonized beads (Figure 8d). Analysis of merged fluorescence and optical microscopy images confirmed that macrophages phagocytosed the opsonized polystyrene beads. Phagocytic efficiency of M1 cells was significantly greater than that of M0 cells and cells cultured with Li/Sr‐BGs. Similarly, the phagocytic indices of Li/Sr‐BG‐treated cells were significantly lower than that of M1 cells. In contrast, the phagocytic activity of cells treated with Sr‐BGs was not different from that of M1 cells (Figure S4).

The effect of Li/Sr‐BGs on the levels of inflammation‐related proteins in macrophages was evaluated by western blotting (Figure 8e). Cells cultured with Li/Sr‐BGs showed significant increases in p‐GSK‐3β levels compared with M0 and M1 cells on day 3. In contrast, the levels of p‐STAT3 in cells treated with Li/Sr‐BGs were significantly lower than that in M1 cells.

## 4 Discussion

Phosphate‐based BGs have high solubility, which enhances their bioactivity and prevents long‐term retention in the body. However, there are potential risks associated with the rapid release of high concentrations of ions from BGs because they can induce cytotoxicity and adverse reactions [27, 28]. Therefore, precise control of ion release is crucial for achieving favorable effects of BGs. In this study, we aimed to achieve a high concentration of Li^+^ release within a few days to exert anti‐inflammatory effects, while ensuring that Sr^2+^ is not released in an initial burst and is released at optimal concentrations over several months to promote bone regeneration in a manner that follows the pathological stages.

The ion release profiles of the experimental BGs were controlled by adjusting the Al content ratio. While both Sr‐BGs and Li/Sr‐BGs released Sr^2+^ over the long term, an increase in the Al content ratio resulted in a gradual extension of the time required to reach the maximum Sr^2+^ concentration. Phosphate‐based BGs contain numerous phosphorous‐oxygen‐phosphorous (P‐O‐P) bonds [29, 30, 31]. However, when Al ions are incorporated into the phosphate glass network, P‐O‐P bonds are disrupted and P‐O‐Al bonds are formed, which are more stable than P‐O‐P bonds [32, 33]. The incorporation of Al ions into glass networks slightly increases the number of octahedrally coordinated Al ions, thereby strengthening the ionic cross‐linking between the networks and enhancing the chemical durability of BGs [34]. In addition, Sr^2+^ possesses a higher cation field strength compared with Li^+^ [35]. As the cation field strength increases, the P‐O‐P bonds within the glass network shorten, enhancing the chemical durability of BGs. Sr‐BG was therefore considered to release Sr^2+^ in a more prolonged and stable manner than Li‐BG. Therefore, we considered that Sr^2+^ release could be regulated by adjusting the amount of Al added to the phosphate‐based BG to precisely vary its chemical durability.

Each Li/Sr‐BG showed a similar Li^+^ release profile and maintained a released concentration above 1.25 mm for up to 7 days, which is effective in exerting an anti‐inflammatory effect [36]. The release profiles of Li^+^ from Li/Sr‐BGs were similar to that of Li‐BG, indicating that mixing with Sr‐BGs had little influence on Li^+^ release. These results indicated that Li/Sr‐BG, prepared by mixing Li‐BG and Sr‐BG, exhibited a stepwise ion release profile; Li^+^ was released rapidly, followed by the sustained release of Sr^2+^.

Next, we evaluated the effects of Li/Sr‐BGs on BMSCs and demonstrated that osteoblast differentiation was promoted by Li/Sr‐Al0 and Li/Sr‐Al2. Aimaiti et al. reported that the osteoblastic differentiation of MSCs was enhanced in the presence of 25–500 µm Sr^2+^ [37]. Li/Sr‐Al0 and Li/Sr‐Al2 released higher concentrations of Sr^2+^ within this range than the other Li/Sr‐BGs, indicating that osteoblast differentiation was promoted by treatment with Li/Sr‐Al0 and Li/Sr‐Al2. In addition, Sr‐BGs showed the same results as Li/Sr‐BGs. These results therefore indicate that the bone regenerative effect of Li/Sr‐BGs is mediated by Sr‐BGs. Calcineurin/NFATc1 signaling primarily responds to Ca and plays a critical role in the osteoblastic differentiation of MSCs [38, 39, 40]. Sr binds to Ca‐sensing receptors on the plasma membrane and activates calcineurin, which leads to the dephosphorylation of NFATc1. This allows its nuclear translocation and enhances the transcriptional activity of osteogenic marker genes [41, 42, 43]. We showed that BMSCs cultured with Li/Sr‐Al0 or Li/Sr‐Al2 have increased levels of calcineurin and NFATc1, indicating that Sr^2+^ from Li/Sr‐BGs activated calcineurin/NFATc1 signaling to promote osteoblastic differentiation of BMSCs.

We then further evaluated the effect of Li/Sr‐BGs onht osteoclast maturation. Osteoclasts without BG application formed multinucleated giant cells with actin‐rich structures called actin rings, a characteristic feature of osteoclasts [44], whereas Li/Sr‐BG treatment suppressed cell fusion and expression of osteoclast markers. Schumacher et al. reported that the presence of 0.1–10 mm Sr^2+^ suppressed osteoclast maturation, including suppression of typical osteoclast morphology, and reduced osteoclast marker expression levels [45]. Li/Sr‐Al0 and Li/Sr‐Al2 maintained the Sr^2+^ concentration within this range for more than 15 days, indicating that osteoclast maturation was inhibited by Li/Sr‐Al0 and Li/Sr‐Al2. During the differentiation of macrophages into osteoclasts, NFATc1 activation is regulated by RANKL through the activation of NF‐κB and c‐Fos [38, 46]. In NF‐κB/c‐Fos/NFATc1 signaling, Sr inhibits NF‐κB activity, resulting in NFATc1 inactivation [47, 48]. The levels of these proteins were reduced in osteoclasts cultured with Li/Sr‐BGs, indicating that Sr from Li/Sr‐BGs inhibited osteoclast maturation by inactivating NF‐κB/c‐Fos/NFATc1 signaling.

M1 macrophages were examined to evaluate the anti‐inflammatory effects of Li/Sr‐BGs. M1 macrophages treated with Li/Sr‐BGs demonstrated enhanced cell proliferation. In addition, when M1 macrophages were cultured with Li/Sr‐BGs, the expression of inflammatory markers and phagocytic activity decreased, while the expression of anti‐inflammatory markers increased. M1 macrophages exhibit lower proliferative capacity compared with M0 and M2 macrophages [49]. These results therefore indicate that Li/Sr‐BGs can suppress the activity of M1 macrophages and promote their polarization toward M2 macrophages. GSK‐3β, a serine/threonine kinase involved in the Wnt/β‐catenin pathway, promotes the expression of inflammation‐related genes by phosphorylating STAT3 [50, 51]. Li is a direct inhibitor of GSK‐3β by increasing the phosphorylation of serine 9 [52, 53]. Non‐phosphorylated GSK‐3β promotes inflammation by phosphorylating STAT3; therefore, the anti‐inflammatory effects of Li are exerted through the indirect suppression of STAT3 activation [54, 55]. When M1 macrophages were cultured with Li/Sr‐BGs, the level of p‐GSK‐3β increased, while the level of p‐STAT3 decreased. These results indicate that Li^+^ from Li/Sr‐BGs suppressed M1 macrophage activity by promoting GSK‐3β phosphorylation. However, Sr^2+^ can exhibit anti‐inflammatory effects at concentrations between 2.5 and 10 mm [56]. The release of Sr^2+^ from Li/Sr‐BGs was insufficient to reach this range, indicating that the anti‐inflammatory effects of Li/Sr‐BGs were mediated by Li^+^. We previously reported that Li‐BG suppressed M1 macrophage activity and exerted anti‐inflammatory effects in vivo [7]. Therefore, these results indicate that Li/Sr‐BGs possess anti‐inflammatory properties in vivo.

The duration of Sr^2+^ activity is a crucial factor in promoting bone regeneration [57]. The degradation of Li/Sr‐BGs observed in this study is based on in vitro experiments and may not necessarily reflect their behavior in vivo; therefore, further investigation is warranted. However, we believe that effective bone formation can be achieved by tailoring the use of each Li/Sr‐BG based on material design and precise control of Sr^2+^ release.

## 5 Conclusions

In this study, precise control of Li^+^ and Sr^2+^ release was achieved by adjusting the Al content ratio in phosphate‐based BGs. Furthermore, advanced smart bioactive BG composites that release Li^+^ and Sr^2+^ in a stepwise manner were successfully developed by mixing Li‐BG and Sr‐BG. Li/Sr‐BGs have great promise for the fabrication of novel bone regenerative biomaterials that follow the bone regeneration process and facilitate rapid and efficient regeneration by suppressing inflammation at an early stage and supporting long‐term hard tissue formation.

## Conflicts of Interest

The authors declare no conflict of interest.

## Supporting information




**Supporting File 1**: mabi70040‐sup‐0001‐SuppMat.docx.

## Data Availability

The data that support the findings of this study are available from the corresponding author upon reasonable request.
