# Supplementary material for: Fabrication of Lithium/Strontium‐Releasing Smart Bioactive Glasses with Anti‐Inflammatory and Osteogenic Effects Tailored to Pathological Stages
Source: Macromol Biosci. 2025 Jun 29;25(11):e00268. doi: 10.1002/mabi.202500268 (PMC12617691; doi:10.1002/mabi.202500268)
Supplement: Supplementary file 1 — Supporting File 1: mabi70040‐sup‐0001‐SuppMat.docx. [file MABI-25-e00268-s001.docx]

Supporting Information

Fabrication of lithium/strontium-releasing smart bioactive glasses with anti-inflammatory and osteogenic effects tailored to pathological stages

Hirohiko Sakai, Jun-Ichi Sasaki*, Haruaki Kitagawa, Gabriela L. Abe,

Tomoki Kohno, Naoya Funayama and Satoshi Imazato

**
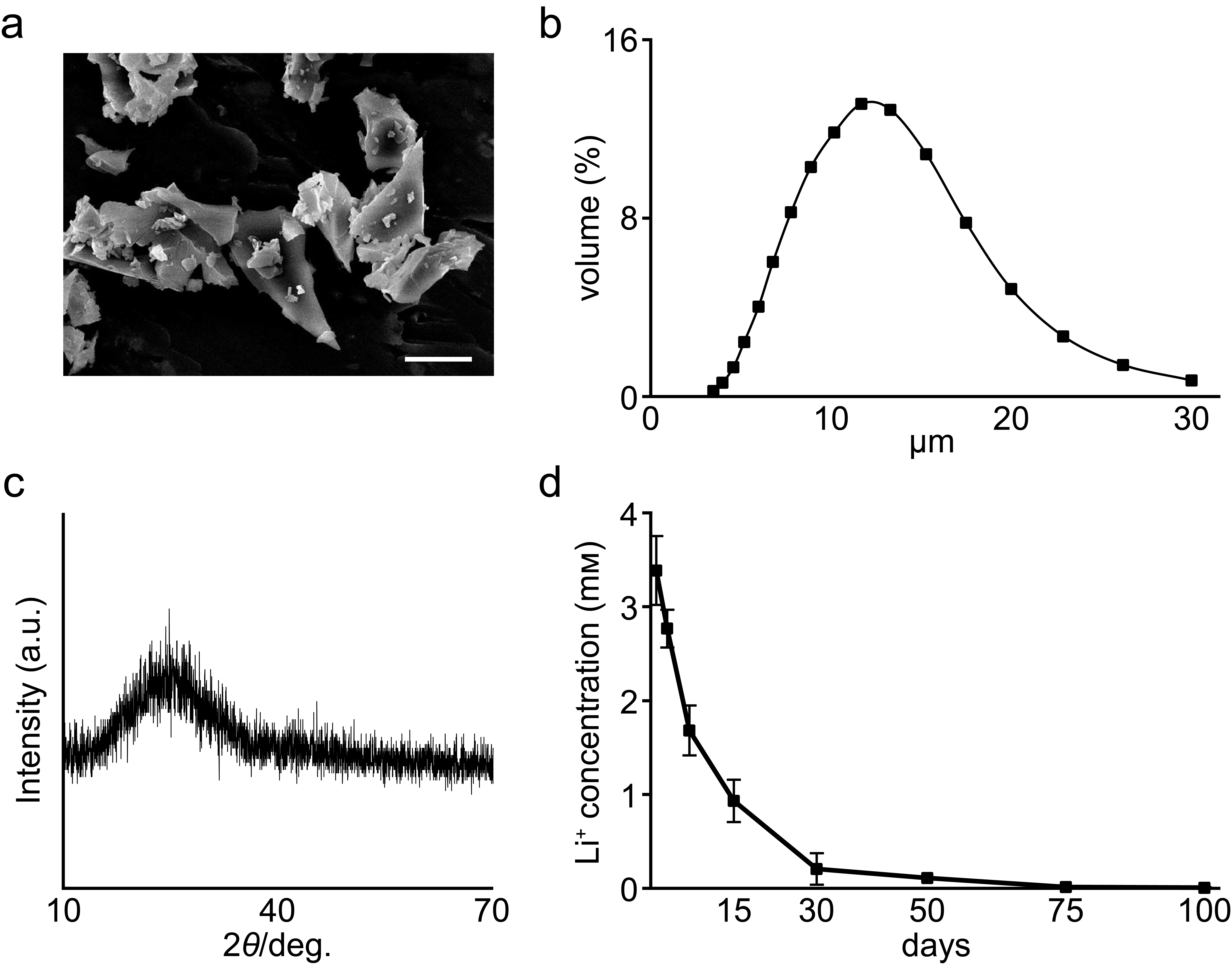
**

**Figure S1.** Characteristics of Li-BG. (a) SEM image and (b) size distribution analysis showed Li-BG particles to have an irregular polygonal shape with a major axis of approximately 10 µm. Scale bar = 10 µm. (c) X-ray diffraction pattern of Li-BG showed a broad band, indicating an amorphous phase. (d) Li^+^ release profile of Li-BG immersed in alpha-minimum essential medium exhibited an initial burst of release, reaching approximately 3.0 mᴍ on day 1, followed by a gradual decline over a period of up to 100 days. Mean ± SD, *n* = 4.


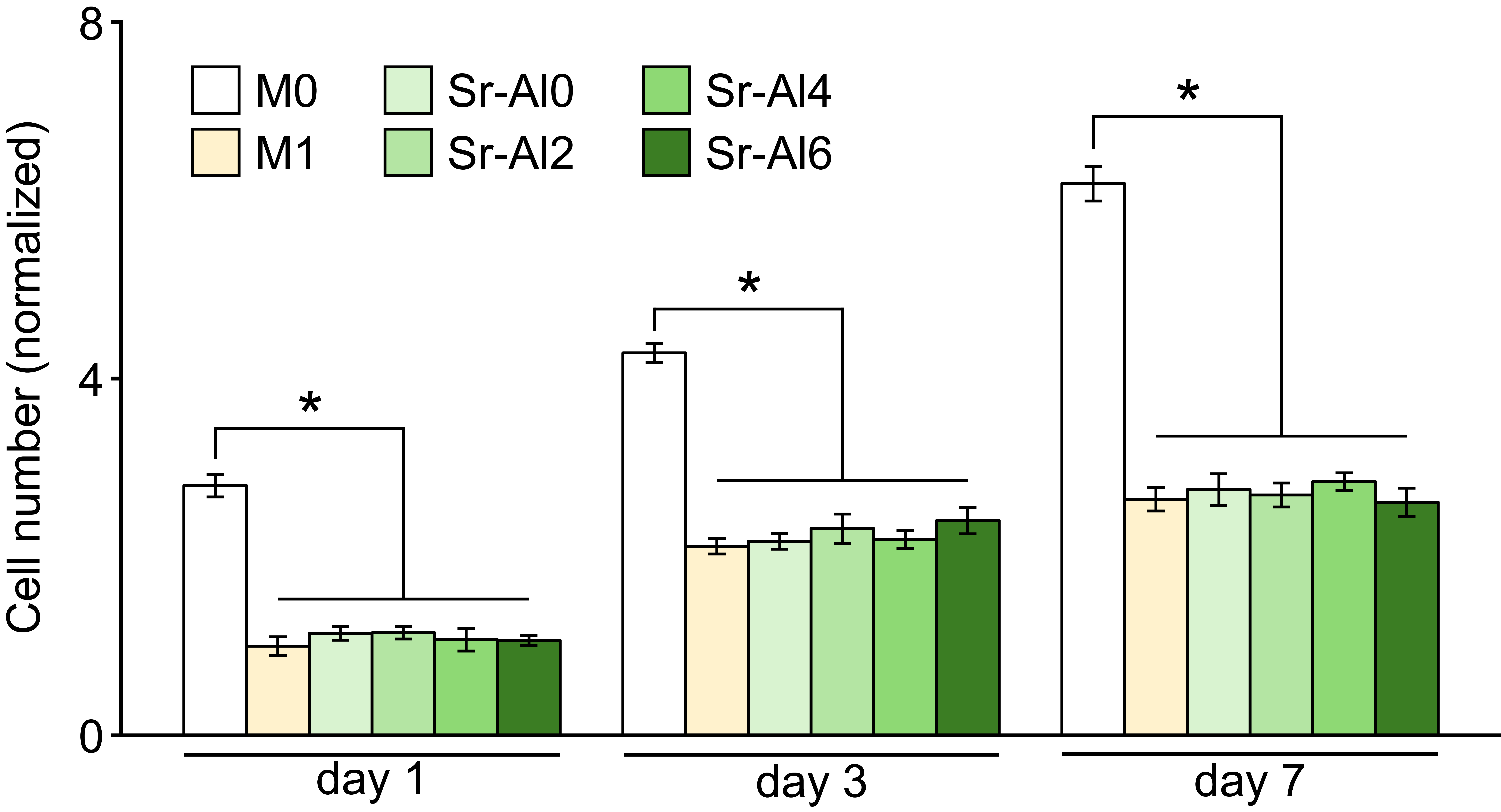


**Figure S2.** Effects of Sr-BGs on macrophage proliferation. The number of proliferative M1 macrophages cultured with Sr-BGs (Sr-Al0–Sr-Al6) was not different from that of M1 macrophages cultured without BG treatment (M1). M0 macrophages cultured without lipopolysaccharide stimulation and Sr-BGs are presented as M0. Asterisks indicate significant differences between groups (*p* < 0.05). Mean ± SD, *n* = 6.


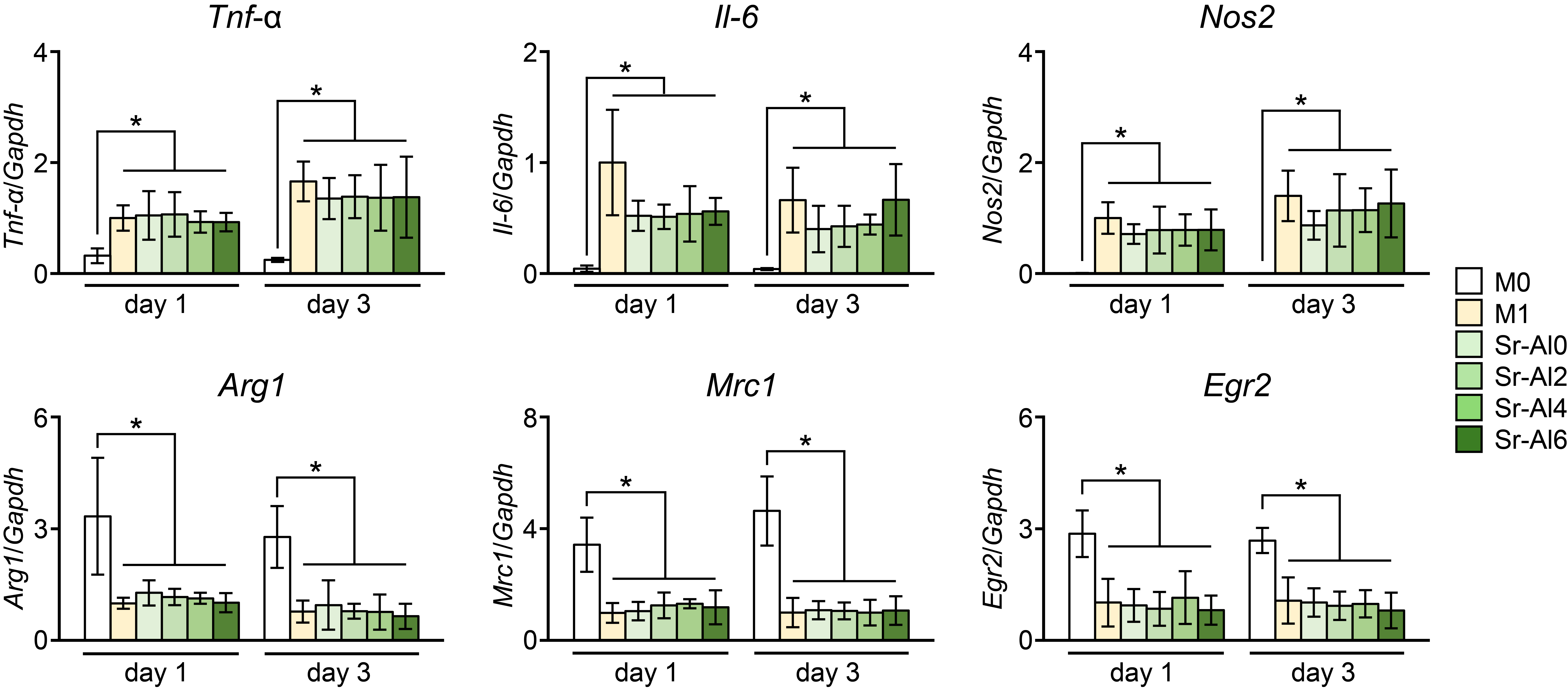


**Figure S3.** mRNA levels of inflammatory-related markers in macrophages cultured with Sr-BGs. The expression levels of both inflammatory (*Tnf-α*, *Il-6*, *Nos2*) and anti-inflammatory (*Arg1*, *Mrc1*, *Egr2*) genes in Sr-BGs-treated cells (Sr-Al0–Sr-Al6) were not different compared with those in M1 macrophages (M1) at day 1 and day 3. M0 macrophages cultured without lipopolysaccharide stimulation and Sr-BGs are presented as M0. Asterisks indicate significant differences between groups (*p* < 0.05). Mean ± SD, *n* = 6.

**
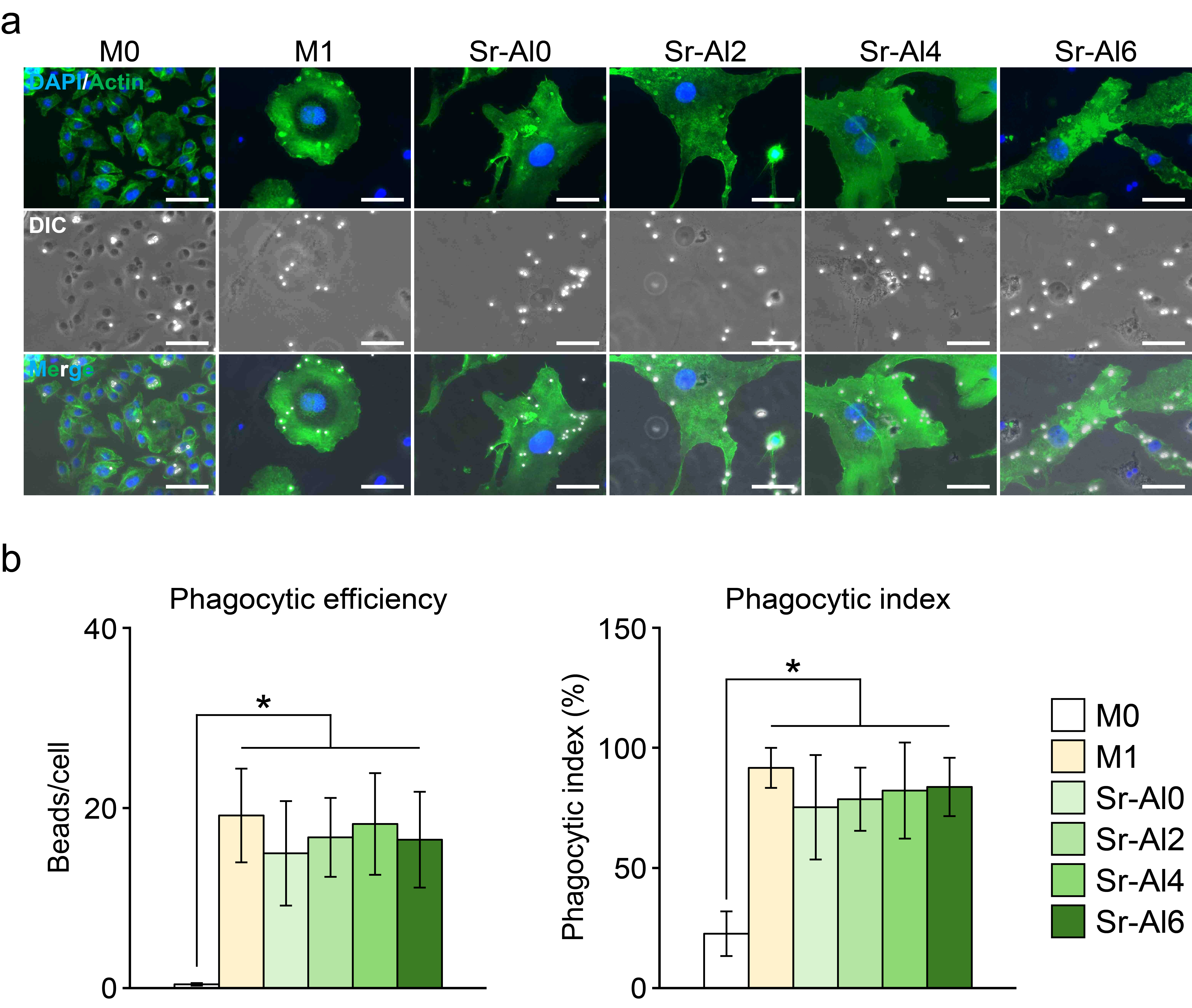
**

**Figure S4.** The effect of Sr-BGs on phagocytic activity of macrophages. (a) Photomicrographs of macrophages engaged in phagocytosis and (b) semi-quantitative analyses of phagocytic activity. The phagocytic activity of cells treated with Sr-BGs (Sr-Al0–Sr-Al6) for 1 day was not different from that of M1 macrophages without BG application (M1). M0 macrophages cultured without both lipopolysaccharide stimulation and Sr-BGs are presented as M0. Asterisks indicate significant differences between groups (*p* < 0.05). Mean ± SD, *n* = 6. Scale bars: 30 µm.
